# Supplementary material for: How to catch a shear band and explain plasticity of metallic glasses with continuum mechanics
Source: Nat Commun. 2024 Jul 3;15:5601. doi: 10.1038/s41467-024-49829-2 (PMC11222483; doi:10.1038/s41467-024-49829-2)
Supplement: Supplementary file 1 — Supplementary Information [file 41467_2024_49829_MOESM1_ESM.docx]

Supplementary Material

**How to catch a shear band and explain plasticity of metallic glasses with continuum mechanics.**

O. Glushko^1^, R. Pippan^2^, D. Sopu^2^, C. Mitterer^1^, J. Eckert^1,2^

^1^ Department of Materials Science, Montanuniversität Leoben, Franz-Josef Straße 18, 8700 Leoben, Austria

^2^ Erich Schmid Institute of Materials Science, Austrian Academy of Sciences, Jahnstraße 12, 8700 Leoben, Austria


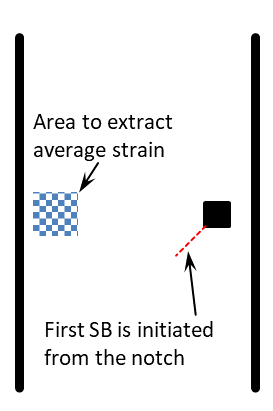


Supplementary Fig. 1. Estimation of local strain values at SB initiation event. At the loading step when the first SB is initiated from the corner of the square notch, the strain values (ε_xx_ and ε_yy_) are averaged over the marked area located symmetrically with respect to the square notch. The average Mises strain from this area is then calculated and multiplied by a factor of 3 (corresponding to SCF) to estimate the local strain at the square notch.
