# Supplementary material for: How to catch a shear band and explain plasticity of metallic glasses with continuum mechanics
Source: Nat Commun. 2024 Jul 3;15:5601. doi: 10.1038/s41467-024-49829-2 (PMC11222483; doi:10.1038/s41467-024-49829-2)
Supplement: Supplementary file 3 — Description of Additional Supplementary Information [file 41467_2024_49829_MOESM3_ESM.pdf]

Description of Additional Supplementary Files

Supplementary Video 1. Evolution of εxx, εyy, and εxy local strain components with increasing global strain corresponding to the case shown in Fig. 2a-2d.

Supplementary Video 2. Evolution of εxx, εyy, and εxy local strain components with increasing global strain corresponding to the case shown in Fig. 3a-3c

Supplementary Video 3. Evolution of εxx, εyy, and εxy local strain components with increasing global strain corresponding to the case shown in Fig. 4a-4c.

Supplementary data. Raw SEM images (ZIP archive) used for DIC analysis of the case shown in Fig. 2a-2d.
