# Supplementary figures and images for: How to catch a shear band and explain plasticity of metallic glasses with continuum mechanics
Source: Nat Commun. 2024 Jul 3;15:5601. doi: 10.1038/s41467-024-49829-2 (PMC11222483; doi:10.1038/s41467-024-49829-2)

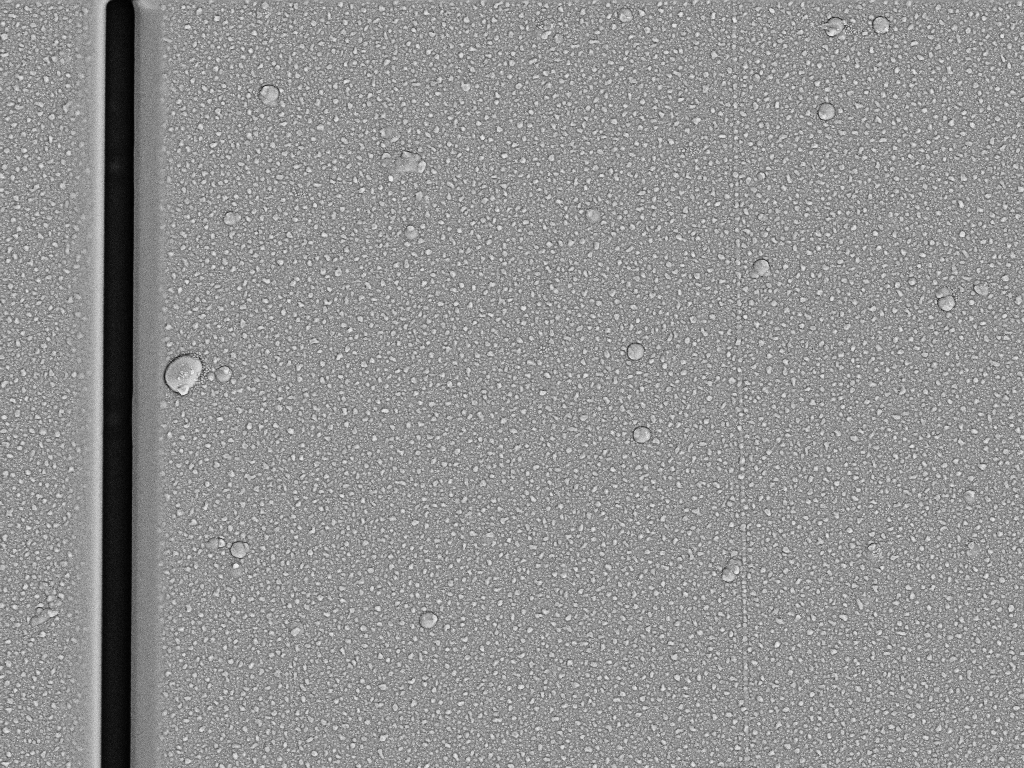

Supplement: Supplementary file 7 — Source Data [file 41467_2024_49829_MOESM7_ESM.zip › PdSi-S20-FIB1-b2-00.tif]

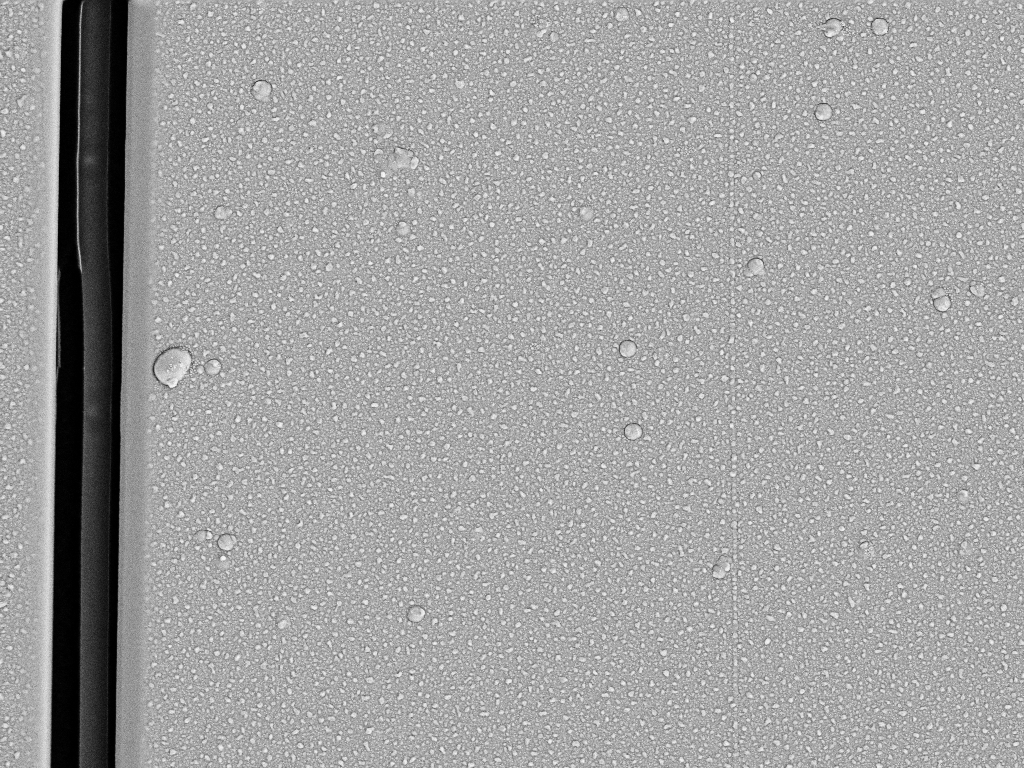

Supplement: Supplementary file 7 — Source Data [file 41467_2024_49829_MOESM7_ESM.zip › PdSi-S20-FIB1-b2-01.tif]

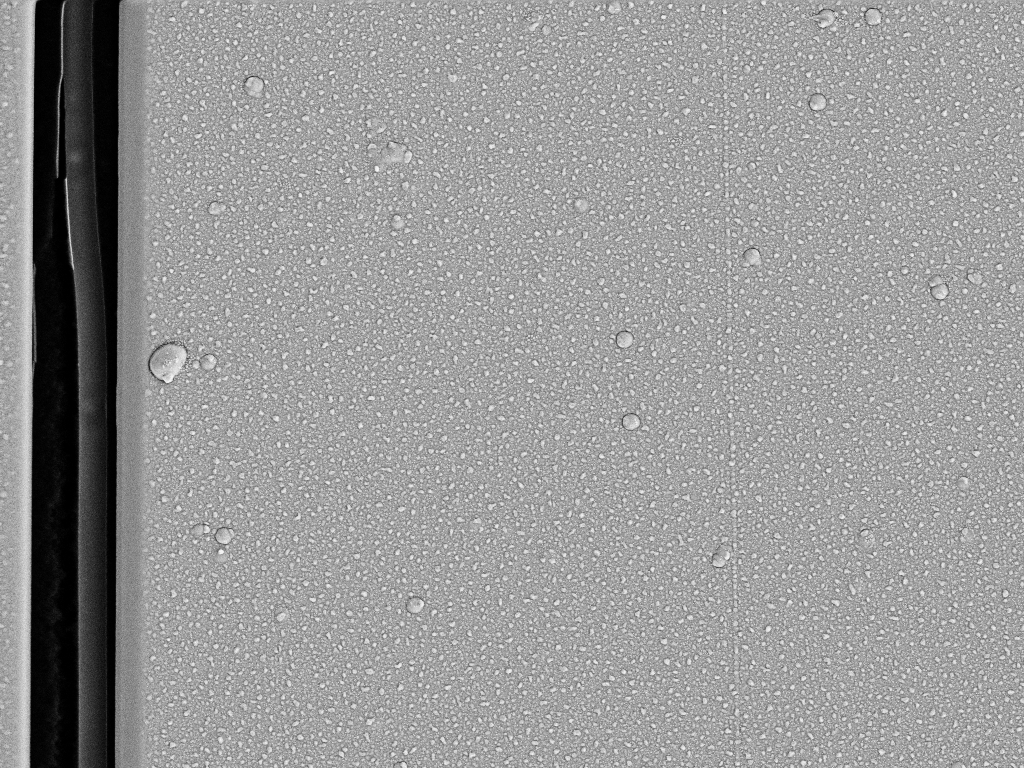

Supplement: Supplementary file 7 — Source Data [file 41467_2024_49829_MOESM7_ESM.zip › PdSi-S20-FIB1-b2-02.tif]

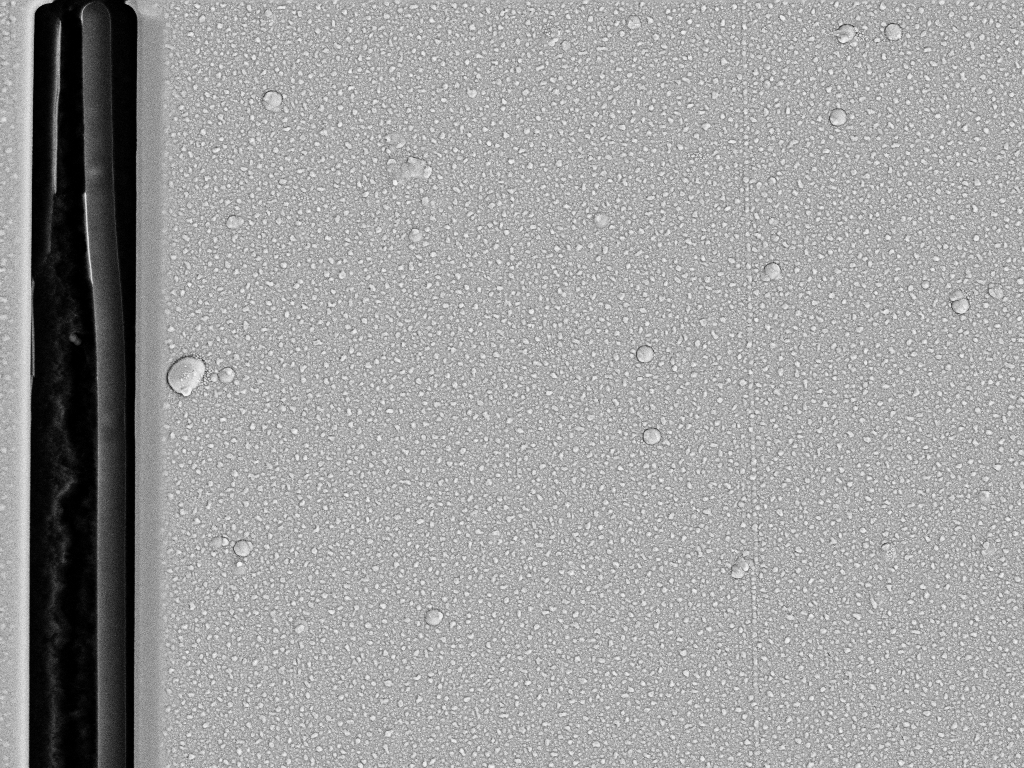

Supplement: Supplementary file 7 — Source Data [file 41467_2024_49829_MOESM7_ESM.zip › PdSi-S20-FIB1-b2-03.tif]

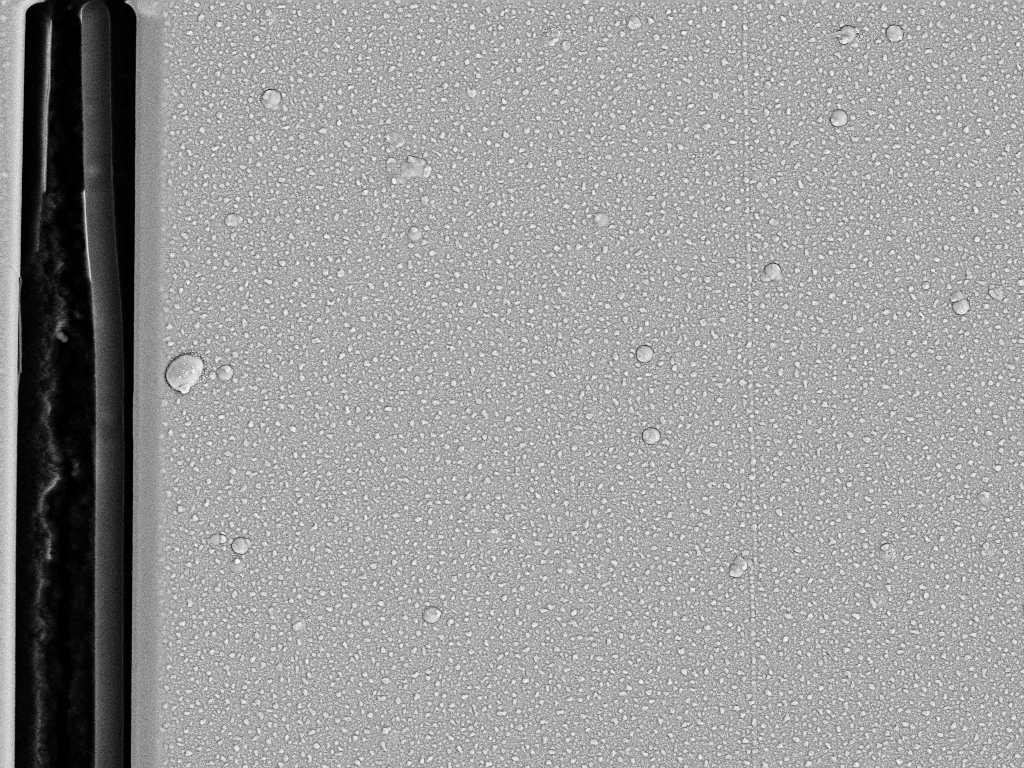

Supplement: Supplementary file 7 — Source Data [file 41467_2024_49829_MOESM7_ESM.zip › PdSi-S20-FIB1-b2-04.tif]

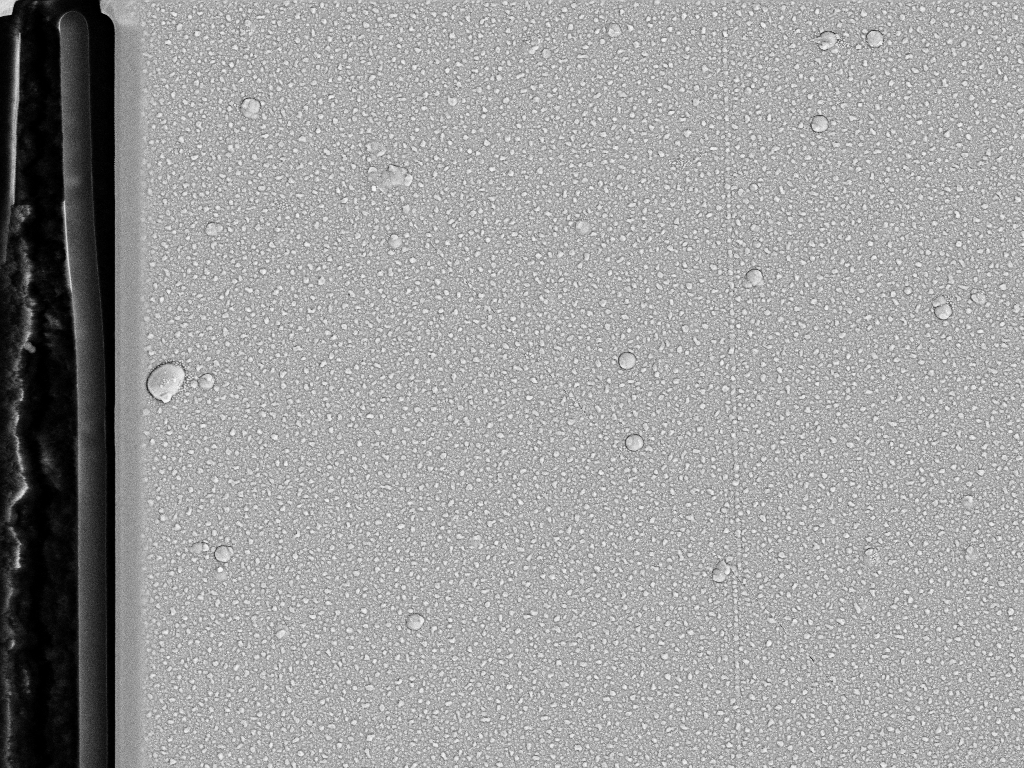

Supplement: Supplementary file 7 — Source Data [file 41467_2024_49829_MOESM7_ESM.zip › PdSi-S20-FIB1-b2-05.tif]

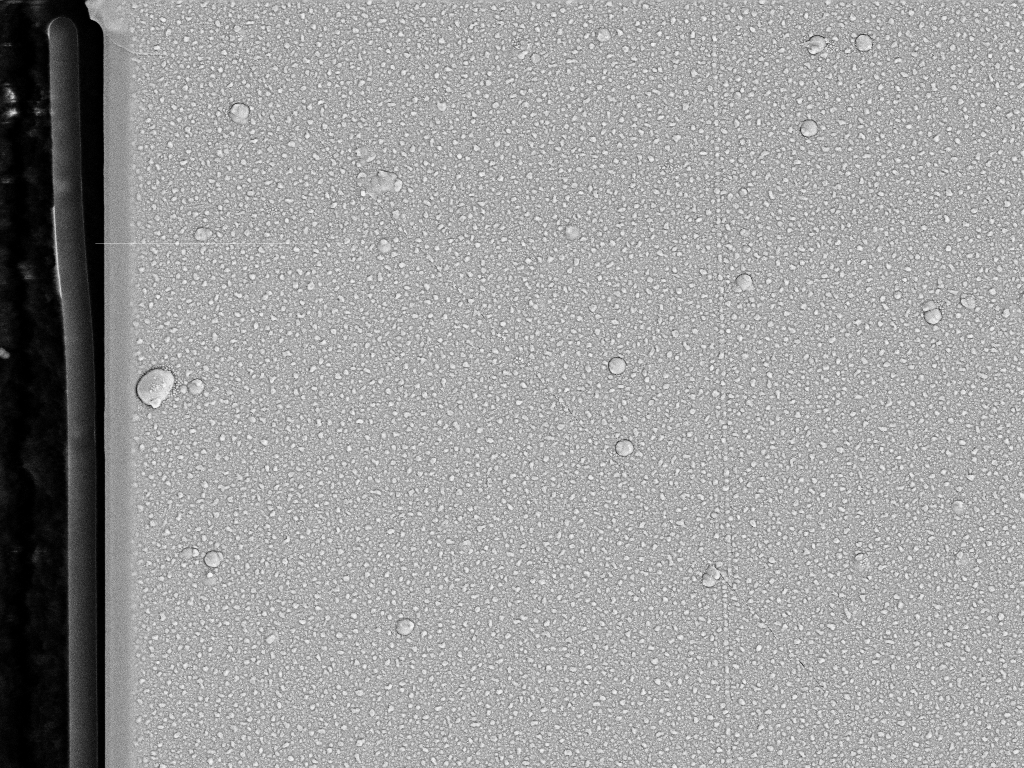

Supplement: Supplementary file 7 — Source Data [file 41467_2024_49829_MOESM7_ESM.zip › PdSi-S20-FIB1-b2-06.tif]

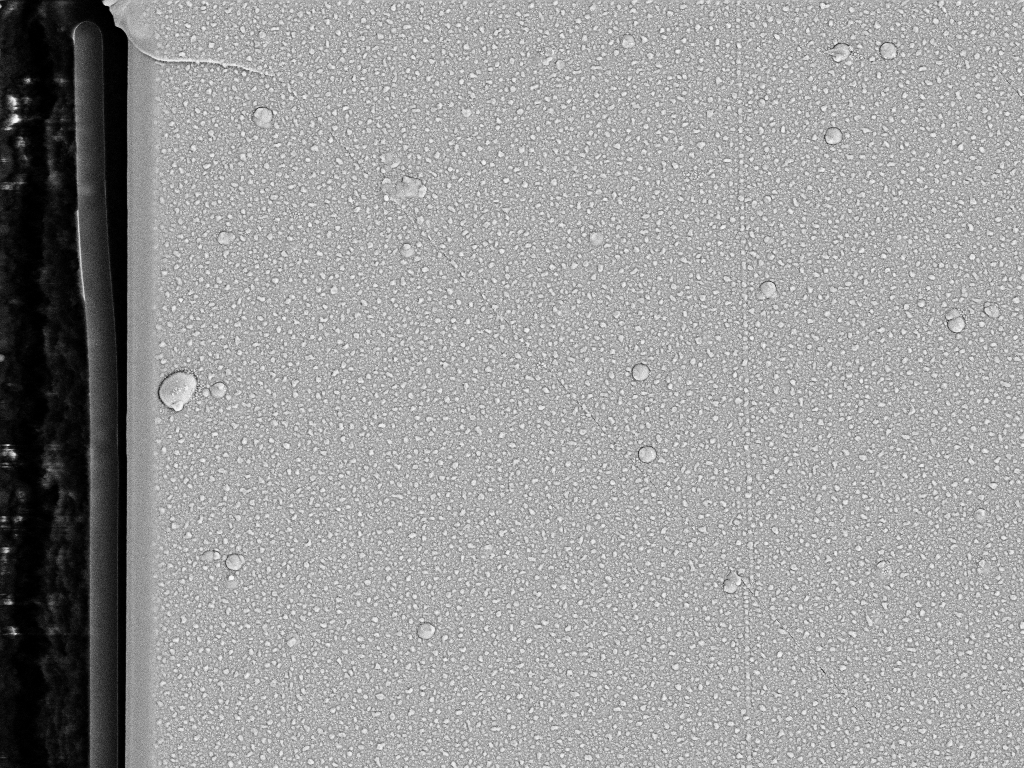

Supplement: Supplementary file 7 — Source Data [file 41467_2024_49829_MOESM7_ESM.zip › PdSi-S20-FIB1-b2-07.tif]

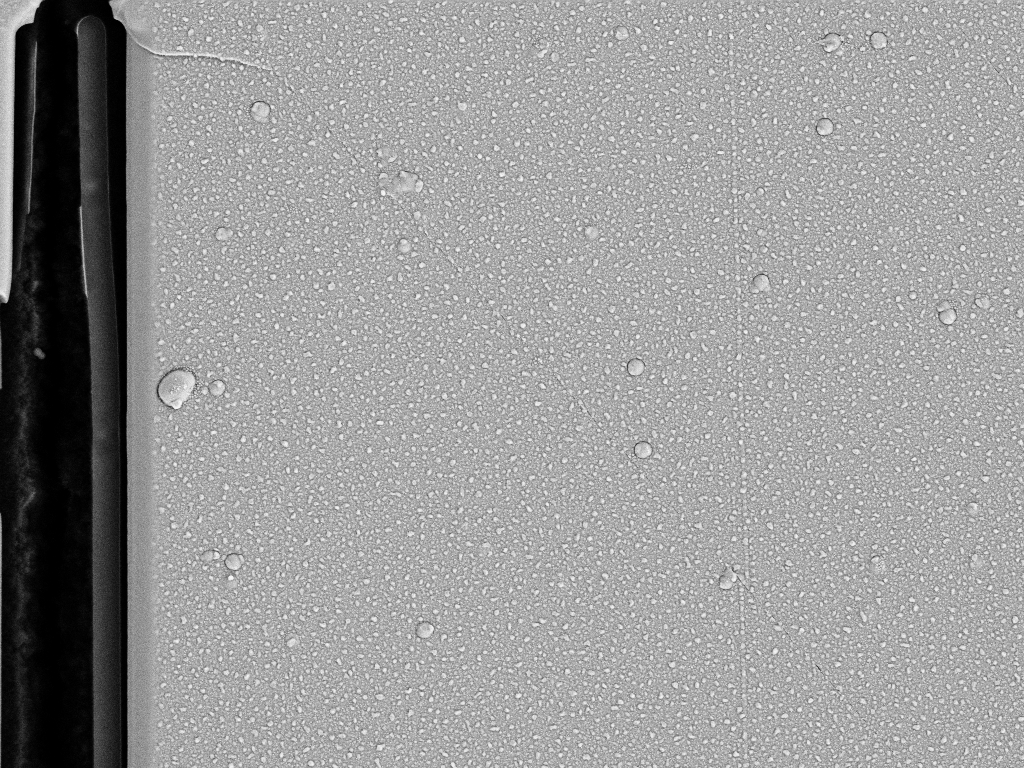

Supplement: Supplementary file 7 — Source Data [file 41467_2024_49829_MOESM7_ESM.zip › PdSi-S20-FIB1-b2-08.tif]

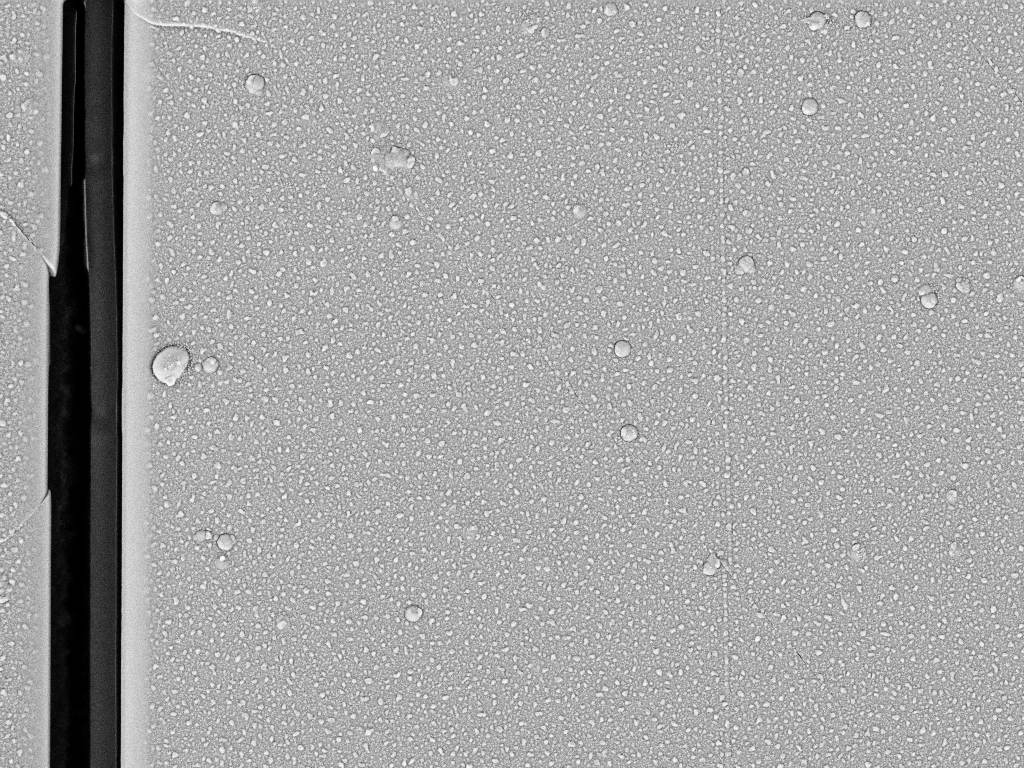

Supplement: Supplementary file 7 — Source Data [file 41467_2024_49829_MOESM7_ESM.zip › PdSi-S20-FIB1-b2-09.tif]
